# Intermediate Phenotypes Identify Divergent Pathways to Alzheimer's Disease

**DOI:** 10.1371/journal.pone.0011244

**Published:** 2010-06-21

**Authors:** Joshua M. Shulman, Lori B. Chibnik, Cristin Aubin, Julie A. Schneider, David A. Bennett, Philip L. De Jager

**Affiliations:** 1 Program in Translational NeuroPsychiatric Genomics, Department of Neurology, Brigham and Women's Hospital, Boston, Massachusetts, United States of America; 2 Harvard Medical School, Boston, Massachusetts, United States of America; 3 Program in Medical and Population Genetics, Broad Institute, Cambridge, Massachusetts, United States of America; 4 Department of Pathology, Rush University Medical Center, Chicago, Illinois, United States of America; 5 Rush Alzheimer's Disease Center, Department of Neurological Sciences, Rush University Medical Center, Chicago, Illinois, United States of America; University of Muenster, Germany

## Abstract

**Background:**

Recent genetic studies have identified a growing number of loci with suggestive evidence of association with susceptibility to Alzheimer's disease (AD). However, little is known of the role of these candidate genes in influencing intermediate phenotypes associated with a diagnosis of AD, including cognitive decline or AD neuropathologic burden.

**Methods/Principal Findings:**

Thirty-two single nucleotide polymorphisms (SNPs) previously implicated in AD susceptibility were genotyped in 414 subjects with both annual clinical evaluation and completed brain autopsies from the Religious Orders Study and the Rush Memory and Aging Project. Regression analyses evaluated the relation of SNP genotypes to continuous measures of AD neuropathology and cognitive function proximate to death. A SNP in the zinc finger protein 224 gene (*ZNF224*, *rs3746319*) was associated with both global AD neuropathology (*p* = 0.009) and global cognition (*p* = 0.002); whereas, a SNP at the phosphoenolpyruvate carboxykinase locus (*PCK1, rs8192708*) was selectively associated with global cognition (*p* = 3.57×10^−4^). The association of *ZNF224* with cognitive impairment was mediated by neurofibrillary tangles, whereas *PCK1* largely influenced cognition independent of AD pathology, as well as Lewy bodies and infarcts.

**Conclusions/Significance:**

The findings support the association of several loci with AD, and suggest how intermediate phenotypes can enhance analysis of susceptibility loci in this complex genetic disorder.

## Introduction

Alzheimer's disease (AD), the most common cause of dementia, leads to progressive loss of memory and other cognitive domains, and is characterized pathologically by the accumulation of extracelluar amyloid plaques and intracellular neurofibrillary tangles. AD likely develops from an interaction of numerous genes along with environmental risk factors, each with modest and incompletely penetrant effects. Linkage studies have identified rare gene mutations as causal in familial, early age-of-onset AD, but these Mendelian variants only explain a small fraction of disease burden in the general population [1]. The identification of susceptibility loci for sporadic, late age-of-onset AD has been more challenging, with numerous reports of candidate gene associations, most of which have not been consistently replicated in follow-up studies [2]–[4]. One notable exception is the *apolipoprotein E* locus (*APOE*): the *ε4* allele is common, increases AD susceptibility 3-fold, and is estimated to explain at least 10% of the population-attributable risk of disease [1]. In addition, the *APOE ε2* allele is a validated AD protective allele though it is less common, and its effect size is more modest than *ε4*.

Genome-wide association (GWA) studies have emerged as a promising approach to identify susceptibility loci in common diseases with complex genetic inheritance, but until recently, most GWA scans in AD have been relatively underpowered, and identified loci have not been consistently replicated [5]–[14]. Increasing sample size is one approach for boosting statistical power, and this strategy has recently led to the identification of several promising new AD susceptibility loci, including *CR1*, *CLU*, and *PICALM* [15], [16]. However, clinical heterogeneity remains a significant confounder of the case/control study design in AD, due to the likely inclusion of dementia cases with multiple pathologies, such as cerebrovascular disease or other neurodegenerative conditions. In addition, since AD develops following a protracted pre-clinical phase consisting of mild symptoms, control groups are susceptible to contamination by latent disease cases. Substantial AD pathology is often present in advanced age, including in those with minimal or no cognitive impairment at death [17]. Subjects with significant pathology but subclinical disease are likely to dilute power in an AD case/control association analysis.

One approach to overcoming these obstacles is to study quantitative intermediate phenotypes. The manifestation of the AD clinical syndrome is the final culmination of a sequence of events beginning with genetic and environmental risk factors that trigger intermediate pathological changes, synapse loss and cell death, and ultimately cognitive decline and dementia. Outcome measures selected more proximally along this causal chain are expected be less confounded and more strongly associated with susceptibility loci. In addition, compared to the dichotomous clinical diagnosis, quantitative intermediate phenotypes can capture more of the underlying heritable trait variation, further enhancing statistical power. Based on this promise, a number of studies have begun to take advantage of intermediate phenotypes for genetic association analysis in AD, including neuropsychiatric test measures [18], MRI imaging data [19], [20], biomarkers from blood and CSF [21], [22], and direct measurements of AD pathology [23]. The latter approach requires access to large study populations with detailed clinical and neuropathologic characterization. The Religious Orders Study and Rush Memory and Aging Project are prospectively following more than 2,300 older persons, all of whom have agreed to annual clinical evaluation and brain donation at death. More than 800 autopsies have been completed to date, and quantitative analyses of amyloid and tangle burden has been performed on nearly 600. In a recent study of *APOE* in this cohort, we found that intermediate cognitive and pathological phenotypes substantially increase power for genetic association analysis [23]. In addition, using neuropathologic phenotypes, the association between *APOE* and cognitive impairment was previously shown to be mediated by a sequential cascade of amyloid plaque formation and subsequent development of neurofibrillary tangle pathology [24], [25]. Therefore, beyond enhancing power for association analysis, intermediate phenotypes hold the additional promise of testing mechanistic hypotheses of gene action.

In this study, we extend our previous work to evaluate several candidate AD susceptibility loci for associations with intermediate phenotypes relevant to AD. Thirty-two candidate SNPs were selected based on their discovery in AD GWA studies and/or evidence from the AlzGene online meta-analyses [2], [26]. SNPs were genotyped in more than 400 subjects with detailed cognitive and pathological data, allowing assessment of genotype relations to quantitative AD pathology and cognitive function proximate to death. We subsequently leveraged the detailed phenotypes available in our cohorts to dissect the functional pathways that link genetic variants to cognitive impairment.

## Methods

### Ethics Statement

Written informed consent and an Anatomic Gift Act were signed by all Religious Orders Study and Rush Memory and Aging project participants after the procedures were fully explained, and both studies were approved by the Institutional Review Board of Rush University Medical Center. The work described in this report was additionally approved by the Institutional Review Boards of the Brigham and Women's Hospital and Massachusetts Institute of Technology.

### Subjects

Clinical and post-mortem data came from participants in the Religious Orders Study and Rush Memory and Aging Project, two longitudinal, epidemiologic clinical-pathologic studies of aging and AD [17]. In both studies, participants without known dementia at baseline agreed to annual detailed clinical evaluation and brain donation at the time of death. Participants in the Religious Orders Study were older Cathololic nuns, priests and brothers from about 40 groups in 12 states across the United States. Subjects in the Rush Memory and Aging Project were older, community-dwelling persons from about 40 retirement communities and subsidized senior housing facilities across northeastern Illinois. Since 1993, more than 2,300 persons agreed to participate in these studies. The overall follow-up rate exceeds 90% of survivors and the overall autopsy rate exceeds 90% of decedents. Of those subjects with completed neuropathologic analyses, and following genotyping quality control filters, 414 persons with genotyping data were available for analysis in February of 2009 when this study was initiated (250 from the Religious Orders Study and 164 from the Rush Memory and Aging Project).

### Clinical evaluation

The clinical diagnoses of dementia and AD were made each year following the recommendations of the joint working group of the National Institute of Neurologic and Communicative Disorders and Stroke and the AD and Related Disorders Association [27], as previously described in detail [28]. Probable AD refers to persons with clinical AD and no other clinical condition contributing to cognitive impairment and possible AD refers to persons meeting inclusion criteria for AD who are thought to have another condition (e.g., stroke) contributing to cognitive impairment. MCI referred to those individuals rated as cognitively impaired by the neuropsychologist but not demented by the examining physician, as previously described [29]. At the time of death, clinical data were reviewed by a neurologist without access to post-mortem data and a summary diagnostic opinion was rendered regarding the most likely clinical diagnosis at the time of death. Level of cognition was based on cognitive testing performed proximate to death. The Religious Orders Study and Rush Memory and Aging Project have 19 cognitive performance tests in common, and use identical analytic procedures to develop summary statistics. Mini-Mental State Examination [30] was used to describe the cohort and one test was used for diagnostic classification purposes only. The remaining 17 tests have been previously described [17]. Tests were converted to *z* scores, using the mean and SD from the baseline evaluation of all participants, and averaged to yield summary measures of global cognition and five cognitive domains: episodic memory, semantic memory, working memory, perceptual speed, and visuospatial ability. Summary measures minimize floor and ceiling effects and other sources of random variability. For the mediation analyses incorporating diagnosis of diabetes, annual clinical evaluations allowed documentation of history of diabetes and use of medications to treat diabetes. Diabetes was determined to be present if the participant was ever taking a medication, such as insulin or an oral hypoglycemic, to treat diabetes, as determined by direct inspection of medication containers, or ever reported a history of diagnosis of diabetes, or both, as previously described [31].

### Neuropathological evaluation

Brain autopsies were performed across the US as previously described [17]. Bielschowsky silver stain was used to visualize neuritic plaques, diffuse plaques, and neurofibrillary tangles in tissue sections from the midfrontal, middle temporal, inferior parietal, and entorhinal cortices, and the hippocampal CA1 sector. The neuropathologic diagnosis of AD was made by a board-certified neuropathologist without access to any clinical data as previously reported [17], [28]. We classified persons as having pathologic AD based on intermediate or high likelihood of AD by National Institute on Aging (NIA)-Reagan criteria using CERAD estimates of neuritic plaque density and Braak staging of neurofibrillary pathology [32]–[34], as previously described [17]. The quantitative composite AD pathology score was based on counts of neuritic plaques, diffuse plaques and neurofibrillary tangles as previously described [35], [36]. Because the means, standard deviations, and ranges of the data varied widely for the pathologic indices, we converted the raw counts to a standard distribution by dividing each person's count by the standard deviation for that particular count and formed a summary measure by averaging the scaled scores. Because the data were skewed, square root of the scaled score was used in analyses. Separate summary measures of neurofibrillary tangles and neuritic and diffuse plaques were also made. Chronic macroscopic cerebral infarctions and alpha-synuclein immunoreactive Lewy bodies were determined as previously described and considered present or absent for analyses [17].

### Genotyping

DNA was extracted from lymphocytes or frozen post-mortem brain tissue. *APOE* genotyping was performed by Agencourt Bioscience Corporation (Beverly, MA) utilizing high throughput sequencing of codon 112 (position 3937) and codon 158 (position 4075) of exon 4 of the *APOE* gene on chromosome 19. In addition to the *APOE ε4* and *ε2* alleles, 32 SNPs were selected for genotyping in our cohort, based on prior evidence from the literature, as of February, 2009. Thus, the more recently discovered *CR1*, *CLU*, and *PICALM* [15], [16] loci were not included in this study, but are the focus of a separate study (Chibnik et al., submitted). The selected SNPs were equally divided between the top results of AD case/control GWA studies [5]–[7], [9], [10]–[14] (16 SNPs) and candidate gene association studies (16 SNPs), which were chosen based on their top ranking in AlzGene meta-analyses [2]. The 32 candidate SNPs were genotyped using matrix-assisted laser desorption-ionization time-of-flight mass spectrometry on a MassARRAY platform (Sequenom). After excluding subjects for failed genotyping exceeding the 10% threshold, 414 individuals remained for subsequent analysis (genotyping rate in these subjects was >99%). All SNP allele frequencies satisfied Hardy-Weinberg equilibrium (p>0.001). Allele frequencies were not significantly different between the Religious Orders Study and Rush Memory and Aging Project subjects, supporting the validity of pooled analyses.

### Statistical Analysis

Given the complementary study designs and similar procedures for data collection and generation of the cognitive and neuropathologic outcome traits, we pooled data from the Religious Orders Study and Rush Memory and Aging Project for our analyses, consistent with numerous prior studies [17], [23]. Genetic association was performed using the PLINK analysis software toolkit [37]. Linear regression was used to evaluate the association of allele genotypes with level of cognition proximate to death in a 2-degree-of-freedom, genotypic test of association, with covariates included for age, gender, and years of education. In order to refine the genetic model, we additionally tested selected SNPs using a 1-degree-of-freedom test to examine for additive, dominant, or recessive allelic effects. These studies were performed using PLINK as well as the R statistical computing program (www.r-project.org). Linear regression modeling in R was used to calculate residual quantitative trait variance explained, and to perform statistical mediation analyses. For the case/control association analysis based on AD clinical diagnosis, logistic regression was performed in PLINK under both additive and genotypic models, and again including covariates for age, gender, and education. All *p*-values reported are unadjusted for multiple hypothesis testing. A Bonferroni-corrected significance threshold of *p*<0.001 was calculated for the 34 SNPs tested for associations with our two primary outcomes, a quantitative measure of global cognitive performance and global AD pathology. Given the high correlation between the pathologic and cognitive traits, applying an adjustment for 68 tests would be overly conservative. Otherwise, the threshold of *p*<0.01 was selected to indicate suggestive statistical evidence of association. All other evaluated phenotypes, including those for AD clinical diagnosis, pathology sub-types, and cognitive domains were considered secondary analyses.

## Results

### Associations with global AD pathology and global cognition

Subject demographics, clinical and neuropathologic diagnoses, cognitive status, and *APOE* genotypes for the cohort analyzed in this study are presented in Table 1. In clinical evaluations proximate to death, of the 414 subjects in our study cohort, 131 (31.6%) had normal cognition, 98 (23.7%) had mild cognitive impairment, and 185 (44.7%) were demented (173 met criteria for possible or probable AD). As expected, a significant proportion (41.5%) of individuals without dementia satisfied NIA-Reagan pathological criteria for intermediate or high likelihood AD.

**Table 1:** Demographic, clinical and pathological characteristics of the study cohort.

**n**	414
**Mean age, y (SD)**	87.1 (6.9)
**Male (%)**	161 (38.9)
**Education, y (SD)**	16.5 (3.6)
**Mini-Mental State Examination (SD)**	21.4 (9.2)
**NINCDS clinical AD (%)**	173 (41.8)
**Mild cognitive impairment (%)**	98 (23.7)
**Diabetes (%)**	78 (18.8)
**APOE ε4 allele present (%)**	121 (29.2)
**APOE ε2 allele present (%)**	64 (15.5)
**NIA-Reagan pathological AD (%)**	236 (57.6)
**Lewy bodies (%)**	77 (18.6)
**Infarcts (%)**	143 (34.5)

We initially tested for associations between each of the 34 polymorphisms and our two primary outcomes, intermediate phenotypes representing a measure of global AD pathologic burden on autopsy and a measure of global cognitive function proximate to death (Table 2). Linear regression models were used to examine the relation of SNP genotypes to the quantitative neuropathologic and cognitive traits in a 2 degree-of-freedom statistical test, adjusting for the effects of age at death, gender, and years of education. As expected, *APOE ε4* was significantly associated with both cognition (*p = *3.4×10^−10^) and AD pathology (*p* = 1.6×10^−24^) in our cohort, whereas an association with *APOE ε2* was only seen for the pathological phenotype (*p* = 9.1×10^−4^). In addition, we found associations with AD intermediate phenotypes for two SNPs, within the zinc finger protein 224 (*ZNF224*) and phosphoenolpyruvate carboxykinase 1 (*PCK1*) genes, both of which were selected for genotyping based on their identification in AD case/control GWA studies [6], [10]. The *ZNF224* SNP (*rs3746319*) was associated with both global cognition (*p* = 0.009) and global AD pathology (*p* = 0.004). In contrast, the *PCK1* SNP (*rs8192708*) was significantly associated with global cognition (*p* = 3.57×10^−4^) but not global AD pathology (*p* = 0.056), suggesting that this locus may influence cognitive impairment through mechanisms other than AD pathology. Besides *APOE ε4*, none of the SNP associations surpass the currently accepted threshold for genome-wide significance (*p*<5.0×10^−8^); however, the association between PCK1 and global cognition exceeds a Bonferroni-corrected significance threshold of p<0.001 for 34 independent tests. Given the high correlation between the pathologic and cognitive traits, applying an adjustment for 68 tests would be overly conservative; however, the PCK1 association still exceeds that standard (*p*<7×10^−4^).

**Table 2:** Relation of candidate AD polymorphisms to intermediate cognitive and pathologic phenotypes.

				Alleles		Global Pathology	Global Cognition
Chr	SNP	Gene	Reference^1^	Minor/Major	MAF^2^	(p^3^)	(p^3^)
1	*Rs4845378*	nicotinic cholinergic receptor (*CHRNB2*)	AlzGene	T/G	0.09	0.794	0.475
	*Rs505058*	lamin A/C (*LMNA*)	Grupe et al. 2007	C/T	0.10	0.366	0.606
	*Rs2584820*	regulator of G protein signaling protein-like 2 (*RGSL2*)	Liu et al. 2007	G/A	0.05	0.823	0.298
	*Rs12044355*	disruptend in schizophrenia 1 (*DISC1*)	Beecham et al. 2009	C/A	0.35	0.959	0.296
2	*Rs1800587*	interleukin-1 Alpha (*IL1A*)	AlzGene	A/G	0.31	0.371	0.724
	*Rs1143634*	interleukin-1 Beta (*IL1B*)	AlzGene	A/G	0.21	0.392	0.907
3	*Rs1049296*	transferrin (*TF*)	AlzGene	T/C	0.15	0.245	0.945
4	*Rs727153*	lecithin retinol acyltransferase (*LRAT*)	Abraham et al. 2008	C/T	0.48	0.157	0.911
9	*Rs7019241*	golgi membrane protein 1 (*GOLM1*)	Li et al. 2008	T/C	0.12	0.512	0.705
	*Rs10868366*	golgi membrane protein 1 (*GOLM1*)	Li et al. 2008	T/G	0.12	0.490	0.730
	*Rs4878104*	death associated protein kinase (*DAPK1*)	Li et al. 2006	T/C	0.37	0.508	0.384
10	*Rs2306604*	transcription factor A, mitochondrial (*TFAM*)	AlzGene	G/A	0.47	0.503	0.742
	*Rs13500*	cholesterol 25-hydroxylase (*CH25H*)	AlzGene	T/C	0.10	0.368	0.579
	*Rs2986017*	calcium homeostasis modulator 1 (*CALHM1*)	AlzGene	A/G	0.26	0.231	0.505
	*Rs600879*	sortilin-related VPS10-containing receptor (*SORCS1*)	AlzGene	A/G	0.11	0.290	0.849
	*Rs1903908*	CG2039140	Grupe et al. 2007	A/G	0.15	0.846	0.912
11	*Rs6265*	brain derived neurotrophic factor (*BDNF*)	AlzGene	A/G	0.19	0.032	0.347
	*Rs1385600*	GRB2-associated binding protein 2 (*GAB2*)	Reiman et al. 2007	C/T	0.18	0.825	0.966
	*Rs2373115*	GRB2-associated binding protein 2 (*GAB2*)	Reiman et al. 2007	T/G	0.18	0.544	0.981
	*Rs2070045*	sortilin-related receptor (*SORL1*)	AlzGene	G/T	0.22	0.182	0.453
	*Rs3824968*	sortilin-related receptor (*SORL1*)	AlzGene	A/T	0.30	0.831	0.683
12	*Rs11610206*	FAM113B	Beecham et al. 2009	C/T	0.08	0.227	0.519
14	*Rs11159647*	14q31.2	Bertram et al. 2008	A/G	0.49	0.968	0.581
17	*Rs1554948*	tyrosine kinase non-receptor 1 (*TNK1*)	Grupe et al. 2007	A/T	0.46	0.441	0.337
	*Rs2471738*	microtubule associated protein tau (*MAPT*)	AlzGene	T/C	0.21	0.533	0.392
	*Rs1800764*	angiotensin converting enzyme (*ACE*)	AlzGene	C/T	0.47	0.451	0.489
19	*Rs3746319*	zinc finger protein 224 (*ZNF224*)	Beecham et al. 2009	A/G	0.16	**0.009**	**0.004**
	*Rs3826656*	CD33	Bertram et al. 2008	G/A	0.20	0.258	0.455
	*Rs3745833*	galinin-related receptor (*GALP*)	Grupe et al. 2007	C/A	0.36	0.024	0.288
20	*Rs1799990*	prion protein (*PRNP*)	AlzGene	G/A	0.33	0.727	0.800
	*Rs8192708*	phosphoenolpyruvate carboxykinase 1 (*PCK1*)	Grupe et al. 2007	G/A	0.12	0.056	**3.57×10^−4^**
23	*Rs5984894*	protocadherin 11 X-linked (*PCDH11X*)	Carrasquillo et al. 2009	A/G	0.48	0.188	0.624
19	*ε4*	apolipoprotein E (*APOE*)		-	0.16	**1.56×10^−24^**	**3.40×10^−10^**
	*ε2*	apolipoprotein E (*APOE*)		-	0.08	**9.15×10^−4^**	0.238

1 SNPs were selected based on AlzGene meta-analyses (ref. 2) or from results of AD GWA studies (refs. 5–7, 9–14).

2 MAF = minor allele frequency.

3 Unadjusted p-values from genotypic regression models, including covariates for age, gender, and years of education.

Although the risk alleles for the associations of both the *ZNF224* and *PCK1* loci with the intermediate phenotypes in our cohort also increase risk of AD diagnosis (Table 3), their effects are opposite to that reported in the original GWA studies [6], [10]. In the case of *ZNF224*, we find that the minor allele, *rs3746319^A^*, is associated with both increased AD pathologic burden and decreased cognitive performance; whereas this variant was protective against AD in the GWA study (G. Beecham and M. Pericak-Vance, personal communication). Similarly, for *PCK1*, the minor allele, *rs8192708^G^*, significantly protected against cognitive decline in our cohort but was in fact the AD risk allele in the original GWA study [2]. Interestingly, two subsequent replication analyses of *rs8192708* documented associations of decreased AD risk with the minor allele, consistent with our findings [4], [38]. Therefore, while the effects of the *ZNF224* and *PCK1* loci on a diagnosis of AD and on intermediate phenotypes are consistent within our study (and in two other *PCK1* replication studies); they are not consistent with the original GWA analyses. In the discussion section, we further address possible explanations for these discrepancies.

**Table 3:** Relation of candidate AD polymorphisms to clinical AD diagnosis.

			MAF 2			p-value 4	
SNP	Gene	A^1^	AD cases	controls	OR (95% CI)^3^	additive	genotypic
*rs4845378*	*CHRNB2*	T	0.08	0.10	0.77 (0.46–1.28)	0.305	0.509
*rs505058*	*LMNA*	C	0.08	0.11	0.81 (0.50–1.33)	0.411	0.571
*rs2584820*	*RGSL2*	G	0.04	0.06	0.63 (0.31–1.26)	0.191	0.589
*rs12044355*	*DISC1*	C	0.35	0.35	1.07 (0.80–1.44)	0.641	0.716
*rs1800587*	*IL1A*	A	0.30	0.31	0.98 (0.72–1.34)	0.896	0.901
*rs1143634*	*IL1B*	A	0.19	0.22	0.87 (0.61–1.26)	0.466	0.729
*rs1049296*	*TF*	T	0.13	0.17	0.76 (0.51–1.14)	0.183	0.285
*rs727153*	*LRAT*	C	0.48	0.49	1.03 (0.77–1.37)	0.860	0.803
*rs7019241*	*GOLM1*	T	0.12	0.12	0.96 (0.60–1.54)	0.871	0.786
*rs10868366*	*GOLM1*	T	0.13	0.13	0.94 (0.59–1.50)	0.804	0.761
*rs4878104*	*DAPK1*	T	0.37	0.37	0.98 (0.74–1.31)	0.907	0.984
*rs2306604*	*TFAM*	G	0.48	0.47	1.01 (0.75–1.36)	0.954	0.354
*rs13500*	*CH25H*	T	0.10	0.10	0.92 (0.58–1.47)	0.731	0.784
*rs2986017*	*CALHM1*	A	0.27	0.26	1.08 (0.78–1.48)	0.645	0.827
*rs600879*	*SORCS1*	A	0.11	0.11	0.91 (0.56–1.45)	0.683	0.71
*rs1903908*	*CG2039140*	A	0.16	0.14	1.14 (0.76–1.71)	0.525	0.707
*rs6265*	*BDNF*	A	0.19	0.18	1.03 (0.71–1.50)	0.885	0.83
*rs1385600*	*GAB2*	C	0.18	0.18	1.03 (0.70–1.51)	0.897	0.73
*rs2373115*	*GAB2*	T	0.18	0.18	0.98 (0.66–1.45)	0.913	0.909
*rs2070045*	*SORL1*	G	0.21	0.22	0.96 (0.67–1.39)	0.846	0.522
*rs3824968*	*SORL1*	A	0.31	0.30	0.98 (0.71–1.35)	0.881	0.891
*rs11610206*	*FAM113B*	C	0.08	0.08	1.02 (0.58–1.79)	0.946	0.985
*rs11159647*	*14q31.2*	A	0.51	0.47	1.17 (0.87–1.57)	0.304	0.588
*rs1554948*	*TNK1*	A	0.46	0.46	0.91 (0.68–1.22)	0.519	0.727
*rs2471738*	*MAPT*	T	0.22	0.20	1.10 (0.77–1.57)	0.606	0.773
*rs1800764*	*ACE*	C	0.49	0.47	1.06 (0.80–1.42)	0.673	0.507
*rs3746319*	*ZNF224*	A	0.19	0.15	1.51 (1.02–2.25)	0.042	**0.008**
*rs3826656*	*CD33*	G	0.20	0.21	0.93 (0.64–1.33)	0.682	0.84
*rs3745833*	*GALP*	C	0.36	0.36	0.94 (0.69–1.28)	0.699	0.845
*rs1799990*	*PRNP*	G	0.32	0.33	0.97 (0.71–1.33)	0.844	0.906
*rs8192708*	*PCK1*	G	0.09	0.14	0.51 (0.32–0.82)	**0.005**	0.011
*rs5984894*	*PCDH11X*	A	0.43	0.52	0.74 (0.58–0.96)	0.021	0.068
*ε4*	*APOE*	-	0.21	0.11	2.67 (1.74–4.11)	**7.0×10^−6^**	**9.63×10^−6^**
*ε2*	*APOE*	-	0.08	0.08	0.76 (0.44–1.32)	0.336	0.876

1 A = minor allele.

2 MAF = minor allele frequency.

3 OR = odds ratio, CI = confidence interval.

4 Unadjusted p-values from logistic regression models, under both additive and genotypic models, including covariates for age, gender, and education.

### Associations with neuritic and diffuse plaques, neurofibrillary tangles, and cognitive subdomains

The global AD pathology score averages the post-mortem density of neuritic and diffuse plaques and neurofibrillary tangles in multiple brain regions; however, we hypothesized that certain AD susceptibility loci might selectively promote one type of pathology, in which case the composite pathologic outcome might dilute statistical power to detect associations. We therefore performed secondary analyses to determine whether any of the candidate SNPs tested demonstrate selective or more robust association signals with separate quantitative measures of plaques or tangle pathology (Table 4). All analyses were again performed using linear regression models to test for associations with SNP genotypes, adjusted for the effects of age, gender, and education. A SNP at the *GALP* locus (*rs3745833*) showed suggestive evidence for association with diffuse plaques (*p* = 0.003), but not with neurofibrillary tangles (*p* = 0.373). In contrast, the *ZNF224* SNP (*rs3746319*) was strongly associated with neurofibrillary tangle burden (*p* = 1.49×10^−4^), whereas no significant association was seen with either neuritic plaque (*p* = 0.018) or diffuse plaque (*p* = 0.290) pathology. Therefore, the association with the tangle subscore is likely the primary driver for the *ZNF224* locus association with global AD pathology (*p* = 0.009), and the composite score appears to dilute statistical power. Interestingly, the *PCK1* SNP (*rs8192708*), which was not associated with the global pathology measure, did show suggestive evidence for association with neuritic plaque pathology (*p* = 0.007); however, this did not appear to explain the strong association with global cognition (*p* = 3.57×10^−4^), as investigated further below.

**Table 4:** Relation of polymorphisms to amyloid plaques and neurofibrillary tangles.

		Amyloid Pathology		Tau Pathology
		Diffuse Plaques	Neuritic Plaques	Neurofibrillary Tangles
SNP	Gene	(p)	(p)	(p)
*rs4845378*	*CHRNB2*	0.554	0.859	0.137
*rs505058*	*LMNA*	0.511	0.307	0.520
*rs2584820*	*RGSL2*	0.199	0.235	0.342
*rs12044355*	*DISC1*	0.801	0.700	0.715
*rs1800587*	*IL1A*	0.265	0.285	0.778
*rs1143634*	*IL1B*	0.367	0.222	0.720
*rs1049296*	*TF*	0.379	0.274	0.521
*rs727153*	*LRAT*	0.041	0.034	0.670
*rs7019241*	*GOLM1*	0.766	0.443	0.686
*rs10868366*	*GOLM1*	0.673	0.509	0.764
*rs4878104*	*DAPK1*	0.673	0.398	0.420
*rs2306604*	*TFAM*	0.345	0.231	0.088
*rs13500*	*CH25H*	0.288	0.349	0.787
*rs2986017*	*CALHM1*	0.748	0.135	0.109
*rs600879*	*SORCS1*	0.523	0.198	0.395
*rs1903908*	*CG2039140*	0.913	0.866	0.680
*rs6265*	*BDNF*	0.117	0.050	0.068
*rs1385600*	*GAB2*	0.571	0.580	0.388
*rs2373115*	*GAB2*	0.739	0.323	0.168
*rs2070045*	*SORL1*	0.052	0.304	0.918
*rs3824968*	*SORL1*	0.794	0.806	0.417
*rs11610206*	*FAM113B*	0.605	0.071	0.438
*rs11159647*	*14q31.2*	0.398	0.616	0.599
*rs1554948*	*TNK1*	0.655	0.254	0.474
*rs2471738*	*MAPT*	0.401	0.769	0.239
*rs1800764*	*ACE*	0.437	0.494	0.627
*rs3746319*	*ZNF224*	0.290	0.018	**1.49×10^−4^**
*rs3826656*	*CD33*	0.769	0.558	0.062
*rs3745833*	*GALP*	**0.003**	0.103	0.373
*rs1799990*	*PRNP*	0.618	0.674	0.801
*rs8192708*	*PCK1*	0.551	**0.007**	0.080
*rs5984894*	*PCDH11X*	0.654	0.166	0.070
*ε4*	*APOE*	**3.74×10^−16^**	**3.44×10^−20^**	**6.5×10^−15^**
*ε2*	*APOE*	**7.16×10^-4^**	**0.001**	0.097

Similar to the approach taken with the pathological phenotypes, we performed secondary analyses to assess whether any of the SNPs showed more robust association with the five cognitive subdomains that comprise the global cognition score. Linear regression was again used to test for association of each SNP with separate quantitative trait outcomes representing episodic memory, semantic memory, working memory, perceptual speed, and visuospatial ability (Table 5). Episodic memory impairment, the most characteristic cognitive deficit of AD, was associated with both the *ZNF224* locus (*p* = 0.003) and the *PCK1* locus (*p* = 3.69×10^−4^). *ZNF224* was additionally associated with decline in visuospatial function (*p* = 0.007), and *PCK1* showed evidence for association with semantic memory impairment (*p* = 0.001).

**Table 5:** Relation of polymorphisms to measures of cognitive performance.

		Episodic Memory	Semantic Memory	Working Memory	Perceptual Speed	Visuospatial Ability
SNP	Gene	(p)	(p)	(p)	(p)	(p)
*rs4845378*	*CHRNB2*	0.315	0.384	0.634	0.959	0.818
*rs505058*	*LMNA*	0.413	0.651	0.624	0.972	0.744
*rs2584820*	*RGSL2*	0.332	0.317	0.311	0.572	0.630
*rs12044355*	*DISC1*	0.548	0.592	0.223	0.700	0.299
*rs1800587*	*IL1A*	0.440	0.360	0.761	0.940	0.766
*rs1143634*	*IL1B*	0.913	0.588	0.978	0.945	0.520
*rs1049296*	*TF*	0.995	0.956	0.702	0.412	0.379
*rs727153*	*LRAT*	0.923	0.904	0.423	0.828	0.951
*rs7019241*	*GOLM1*	0.516	0.515	0.380	0.245	0.489
*rs10868366*	*GOLM1*	0.479	0.641	0.564	0.314	0.490
*rs4878104*	*DAPK1*	0.191	0.715	0.934	0.590	0.797
*rs2306604*	*TFAM*	0.877	0.554	0.444	0.560	0.126
*rs13500*	*CH25H*	0.868	0.407	0.170	0.733	0.143
*rs2986017*	*CALHM1*	0.231	0.881	0.594	0.784	0.816
*rs600879*	*SORCS1*	0.601	0.786	0.955	0.484	0.266
*rs1903908*	*CG2039140*	0.640	0.959	0.714	0.273	0.504
*rs6265*	*BDNF*	0.196	0.375	0.462	0.903	0.913
*rs1385600*	*GAB2*	0.760	0.319	0.865	0.624	0.968
*rs2373115*	*GAB2*	0.957	0.359	0.949	0.702	0.939
*rs2070045*	*SORL1*	0.443	0.952	0.746	0.170	0.357
*rs3824968*	*SORL1*	0.716	0.849	0.898	0.344	0.510
*rs11610206*	*FAM113B*	0.268	0.574	0.572	0.572	0.692
*rs11159647*	*14q31.2*	0.275	0.883	0.749	0.277	0.989
*rs1554948*	*TNK1*	0.494	0.469	0.459	0.278	0.162
*rs2471738*	*MAPT*	0.509	0.401	0.165	0.033	0.202
*rs1800764*	*ACE*	0.475	0.152	0.736	0.153	0.849
*rs3746319*	*ZNF224*	**0.003**	0.031	0.022	0.089	**0.007**
*rs3826656*	*CD33*	0.534	0.310	0.928	0.408	0.767
*rs3745833*	*GALP*	0.212	0.433	0.245	0.956	0.637
*rs1799990*	*PRNP*	0.652	0.952	0.877	0.982	0.650
*rs8192708*	*PCK1*	**3.69×10^−4^**	**0.0010**	0.051	0.012	0.073
*rs5984894*	*PCDH11X*	0.769	0.799	0.460	0.722	0.877
*ε4*	*APOE*	**2.36×10^−12^**	**1.22×10^−7^**	**5.60×10^−6^**	**1.22×10^−4^**	**5.46×10^−4^**
*ε2*	*APOE*	0.130	0.943	0.555	0.126	0.665

### Divergent pathways from genes to cognitive impairment

For the SNPs at the *ZNF224* and *PCK1* loci, we performed additional linear regression analyses to refine the genetic model for the relation with our intermediate phenotypes (additive, dominant, or recessive), better characterize the strength of the observed effects, and develop statistical models to test hypotheses about mechanistic pathways. Our core regression model, consisting of age at death, gender, and years of education, explained 3% and 7% of the variation in our pathological and cognitive traits, respectively. Using the optimal dominant model of inheritance, the *ZNF224* SNP (*rs3746319*) explained an additional 2% (Beta = 0.13, *p* = 0.003) of the residual variance in global AD pathology and 2.1% (Beta = −0.39, *p* = 0.002) of the variance in global cognition (Table 6). We next explored whether the effect of this locus on AD pathology might mediate its association with cognition (Table 7). When a term for global AD pathology was incorporated in our linear regression model, the magnitude of the association between *ZNF224* and global cognition was attenuated by 44% (Beta = −0.22, *p* = 0.05). In our analyses of the neuropathologic subtypes, we found that the association of *ZNF224* on AD pathology appeared to be due to a predominant effect on neurofibrillary tangles. Indeed, when we substituted a term for neurofibrillary tangles instead of the global pathology variable in our regression model, the effect of the *ZNF224* variant on global cognition was reduced by 64%, and was no longer significant (Beta = −0.14, *p* = 0.21), whereas tangles showed a robust association with cognitive impairment (*p*<2×10^−16^). These results are consistent with a sequence of events whereby an effect on the formation of neurofibrillary tangles accounts for the association of the *ZNF224* allele with cognitive function.

**Table 6:** Detailed genotype-phenotype data and statistical modeling.

	Minor Allele Homozygotes			Heterozygotes				Major Allele Homozygotes					
Variant	No. Subjects (Frq.)	Genotype	Mean Score (SD)^1^		No. Subjects (Frq.)	Genotype	Mean Score (SD)^1^	No. Subjects (Frq.)	Genotype	Mean Score (SD)^1^	Beta (SE)	p (Model)^2^	Variance Explained
**Global Pathology**													
*rs3746319 (ZNF224)*	11 (0.03)	AA	0.749 (0.486)		113 (0.28)	AG	0.811 (0.427)	290 (0.70)	GG	0.687 (0.398)	0.13 (0.04)	0.003 (D)	2
*APOE ε2*	2 (0.005)	-	0.388 (0.010)		62 (0.15)	-	0.576 (0.409)	340 (0.84)	-	0.754 (0.409)	−0.20 (0.05)	2.11×10^−4^ (A)	3.1
*APOE ε4*	9 (0.02)	-	0.906 (0.437)		110 (0.27)	-	1.011 (0.357)	285 (0.71)	-	0.609 (0.376)	0.35 (0.04)	<2.0×10^−16^ (A)	18.9
**Global Cognition**													
*rs3746319 (ZNF224)*	11 (0.03)	AA	−0.759 (1.15)		113 (0.27)	AG	−1.19 (1.27)	289 (0.70)	GG	−0.79 (1.15)	−0.39 (0.12)	0.002 (D)	2.1
*rs8192708 (PCK1)*	8 (0.02)	GG	−0.413 (0.670)		81 (0.20)	GA	−0.550 (0.947)	324 (0.78)	AA	−1.00 (1.24)	0.49 (0.13)	1.02×10^−4^ (A)	3.4
*APOE ε2*	2 (0.01)	-	−0.235 (1.11)		62 (0.15)	-	−0.788 (1.21)	339 (0.84)	-	−0.916 (1.19)	0.23 (0.15)	0.12 (A)	0.36
*APOE ε4*	9 (0.02)	-	−1.27 (1.50)		109 (0.27)	-	−1.41 (1.28)	285 (0.71)	-	−0.684 (1.09)	−0.69 (0.11)	4.37×10^−10^ (A)	9

1 Mean quantitative trait outcome measure is reported, square root transformed for global pathology.

2 Associations were tested with additive (A), dominant (D), or recessive models to identify the best fit.

**Table 7:** Distinct pathways of *ZNF224* and *PCK1* association with cognition.

	Model 1 1			Model 2 2			Model 3 3	
	Beta (SE)	p		Beta (SE)	p		Beta (SE)	p
*rs3746319 (ZNF224)*	−0.39 (0.12)	0.002		−0.22 (0.11)	0.05		−0.14 (0.11)	0.213
Pathology Measure	-	-	Global Pathology	−1.27 (0.13)	<2×10^−16^	Neurofibrillary Tangles	−1.40 (0.12)	<2×10^−16^
*rs8192708* (*PCK1*)	0.50 (0.120)	1.02×10^−4^		0.37 (0.12)	0.002		0.31 (0.11)	0.005
Pathology Measure	-	-	Global Pathology	−1.22 (0.13)	<2×10^−16^	Neuritic Plaques	−0.96 (0.10)	<2×10^−16^

1 Core regression model includes terms for age at death, gender, and years of education.

2 Model additionally includes a term for the global AD pathology measure.

3 Model additionally includes a term for either neurofibrillary tangles or neuritic plaques.

In contrast to *ZNF224*, the *PCK1* locus showed a relatively selective association with global cognition, but not with global pathology. For this locus, an additive model of inheritance was a best fit for our data, and *PCK1* explained 3.4% (Beta = 0.49, *p* = 1.02×10^−4^) of the variance in global cognition proximate to death. We next used multiple linear regression to test whether the association of this SNP on cognitive impairment is predominantly independent of AD pathology. Indeed, after inclusion of a model term for global AD pathology, the *PCK1* SNP (*rs8192708*) remained associated with global cognition (Beta = 0.37, *p* = 0.002), despite the strong, independent association between pathology and cognition (Beta = −1.22, *p*<2×10^−16^; Table 7). Given our finding of an association with neuritic plaques, we substituted a model term for neuritic pathology instead of the global pathology variable; however, despite a modest reduction in the effect size, the relation between *PCK1* and cognitive impairment remained significant (Beta = 0.31, *p* = 0.005; Table 7). Besides AD-related pathology, Lewy bodies and infarcts are the two additional brain pathologies most commonly seen in association with age-related cognitive decline [39]. We therefore investigated whether the association of *PCK1* on global cognition might be mediated by either Lewy bodies or infarcts, by including relevant terms into our regression model (Table 8). Again, the association between *PCK1* and global cognition remained significant (Beta = 0.39, *p* = 2.04×10^−4^), and *PCK1* continued to explain 3% of the residual variance in global cognition in our cohort after adjusting for the three most common brain pathologies associated with dementia. Common variation at the *PCK1* locus has also been associated with type 2 diabetes in a number of independent studies [40]–[42]. Since diabetes has also been implicated as a risk factor for age-related cognitive decline [31], we examined whether diabetes mediates the association of *PCK1* with cognitive impairment. However, adjusting for diabetes diagnosis in our linear regression model did not significantly attenuate the association of *PCK1* with global cognition (Beta = 0.370, *p* = 0.001).

**Table 8:** *PCK1* association with cognition is largely independent of AD pathology, infarcts, and Lewy bodies.

	Model 4 1	
	Beta (SE)	p
*rs8192708* (*PCK1*)	0.39 (0.11)	2.04×10^−4^
AD Pathology	−1.24 (0.12)	<2×10^−16^
Infarcts	−0.39 (0.10)	1.75×10^−4^
Lewy bodies	−0.52 (0.11)	3.84×10^−5^

1 Model also includes terms for age, gender, and education.

## Discussion

In this study, by genotyping a panel of loci within two cohorts of subjects with detailed cognitive and neuropathological characterization, we evaluate intermediate phenotypes as a tool for the functional dissection of candidate AD susceptibility loci. Using the extensively validated *APOE* locus, we previously demonstrated that intermediate traits enhance statistical power to detect associations, even in studies of modest sample size. Here, using the same strategy, we present evidence supporting the possible role of two additional loci in influencing age-related cognitive decline and AD neuropathology. Specifically, the *ZNF224* locus is associated with a quantitative measure of global AD pathology, and both *ZNF224* and *PCK1* are associated with a summary measure of global cognition proximate to death. Using separate quantitative traits for each of the predominant AD pathological features, we document associations between *GALP* and *PCK1* and diffuse and neuritic plaque pathology, respectively, whereas *ZNF224* showed a relatively selective association with neurofibrillary tangle pathology. Finally, in a series of statistical mediation analyses, we tested hypotheses about the causal chain of events linking genetic variation in the *ZNF224* and *PCK1* loci with cognitive decline, with strikingly different outcomes. In the case of *ZNF224*, we find that AD pathology, and more specifically, neurofibrillary tangles mediate an association with cognitive impairment. In contrast, we find that the association between *PCK1* and cognition is largely independent of not only AD pathology, but also Lewy bodies, and infarcts, which together comprise the three most common known brain pathologies associated with dementia [39], [43].

Both *ZNF224* and *PCK1* were initially implicated by AD GWA studies; however, neither locus has yet been consistently replicated in subsequent genetic studies, and little is known about their potential mechanism of action in disease pathogenesis. The *ZNF224* locus encodes a Kruppel-associated box-containing zinc-finger protein that is widely expressed, including in the adult brain, and likely functions as a transcriptional repressor [44], [45]. The SNP evaluated in this study, *rs3746319*, encodes a missense mutation causing a Lys to Glu change at position 640, which falls near the C-terminus within one of 19 zinc-finger repeat motifs. However, we do not yet know enough about ZNF224 protein structure and function to speculate further on how this variant might promote neurofibrillary tangle formation and subsequent cognitive impairment, and further investigation will be required to determine if *rs3746319* is the causal variant and whether *ZNF224* is indeed the causal gene. The *PCK1* gene encodes phosphoenolpyruvate carboxykinase 1, which catalyzes the rate-limiting step of gluconeogenesis [46]. The SNP genotyped in our study, *rs8192708*, is also a missense mutation, causing an Ile to Val change at position 267; however, the functional consequences of this change, if any, are not known. *PCK1* variants have also been suggested to be associated with diabetes [40]–[42], and independently, diabetes has been identified as a risk factor for the development of dementia [31]. In our mediation analysis, adjusting for the effect of diabetes diagnosis did not account for the association of *PCK1* and cognitive impairment; however, it is possible that an appropriate intermediate phenotype, such as direct measurements of blood glucose or hemoglobin A1c, might allow detection of mediation. In another study performed in the same cohort, a relation was found between diabetes and infarcts [47]; however, we were also unable to mediate the *PCK1* association by including a model term for cerebral infarctions. Our finding that the *PCK1* association with cognitive decline is not explained by AD pathology, Lewy bodies, or infarcts suggests that this locus might influence additional, unmeasured pathologies. For example, whereas our analyses adjusted for macroscopic infarcts, *PCK1* may instead primarily influence microscopic forms of cerebrovascular injury. Further, while our intermediate pathologic phenotype accounts for amyloid plaques and neurofibrillary tangles, it does not capture levels of soluble, but potentially still neurotoxic, forms of amyloid or tau pathology [48], [49]. Alternatively, variation at *PCK1* might influence one or multiple steps in the cascade of events predicted to occur downstream of amyloid, tangles and other pathologies, such as synapse loss, inflammation, and/or cell death pathways.

Unexpectedly, the variants in *ZNF224* and *PCK1* show opposite directions of allelic effects for association with AD intermediate phenotypes in our cohort compared to their association with AD diagnosis in the initial GWA studies. In other words, the alleles associated with increased AD risk in the initial reports (*rs3746319^G^* and *rs8192708^G^*) are actually protective against cognitive decline in our cohort. Importantly, this discrepancy is not accounted for by our use of intermediate phenotypes, as the *ZNF224* and *PCK1* SNPs show consistent direction of affect on AD diagnosis in our study population (Table 3). Such “flip-flop” associations have been reported with increasing frequency as GWA scans are completed for many common diseases, and replication efforts are subsequently undertaken [50]. Indeed, in the case of *PCK1*, two prior replication studies found evidence that the major allele, *rs8192708^A^*, may increase risk for dementia, consistent with our results suggesting an association between this allele and both cognitive decline and AD [4], [38].

The interpretation of reversals in the direction of variant associations between different study cohorts remains controversial [50]. The most common explanation for such observations are that they are in fact spurious and representative of chance fluctuations around the null hypothesis. However, in our study, the strongly suggestive statistical evidence for the associations between *ZNF224* and *PCK1* with AD intermediate phenotypes makes their arising by chance less likely; and additionally, the reversals of allelic effect are seen with both loci in our analysis. Instead, we propose that differences in subject ascertainment and recruitment are more likely to be responsible for our observations. The Religious Orders Study (ROS) and Rush Memory and Aging Project (MAP), from which our study cohort is based, are prospective, longitudinal studies in which subjects from the community are recruited non-demented at baseline (mean age  = 75 and 79, for ROS and MAP respectively). All cases of clinical AD are therefore incident within our cohort. In contrast, similar to nearly all AD GWA studies performed to date, the initial reports of association with the *PCK1* and *ZNF224* loci come from AD cases recruited from a neurology clinic population with prevalent dementia. In addition, whereas subjects in our study were recruited at approximately similar ages to the GWA cohorts, they were significantly older at the time of last clinical evaluation and autopsy (mean age of death  = 87). Studies with different designs (cross-sectional vs. prospective) and varying methods of subject ascertainment can generate contradictory epidemiological findings, for example due to survival bias. If an AD risk allele is associated with earlier age of dementia onset; it might be under-represented in the prospective cohort, which requires subjects to be non-demented at enrollment; and therefore, might subsequently appear to be a protective allele. “Flip-flop” associations might additionally arise from variation in linkage disequilibrium structure in the genomic region of interest between the cohorts in different studies. In fact, both the *ZNF224* and *PCK1* SNPs fall under modest recombination peaks, based on HapMap data [51]. Although both our study and the GWA analyses were conducted in subjects of European ancestry, it remains possible that sampling variation between two populations of similar ethnicity might lead to the association reversal that we have observed, as recombination could distribute our tag SNP onto haplotypes that are different from that harboring the causal variant [50]. Ultimately, further analysis of both SNPs and fine mapping of each locus in larger study samples will be required to validate both *PCK1*and *ZNF224* as AD susceptibility loci, and resolve which allele may increase risk for disease.

Of the thirty-four SNPs evaluated in our study, both of the loci that we found to be associated with AD intermediate phenotypes were initially identified by GWA studies, suggesting the power of this unbiased approach to identify genes that might be overlooked by prevailing hypotheses of disease biology. Our study was initiated prior to the recent report of two large AD case/control GWA studies which independently identified three new susceptibility genes, *CLU*, *CR1*, and *PICALM* [15], [16]. In a parallel effort, we recently found that *CR1* is associated with age-related cognitive decline in our study cohorts; and further, that this association was mediated by an effect on amyloid pathology (Chibnik et al., submitted). The power of a GWA study design and the types of genes one expects to discover are tightly linked to the selected phenotypic outcome. To the extent possible, the chosen outcome measure should be closely matched to the underlying biology responsible for the heritable trait variation of interest. In autopsy cohort studies of aged individuals in the community setting, most subjects with probable AD demonstrate multiple brain pathologies [52]. Based on our results, we believe that intermediate pathological and cognitive traits have great promise to enhance gene discovery and for functional characterization of loci that emerge from current AD GWA studies.
